# Screening In Vitro Targets Related to Diabetes in Herbal Extracts from Peru: Identification of Active Compounds in *Hypericum laricifolium* Juss. by Offline High-Performance Liquid Chromatography

**DOI:** 10.3390/ijms18122512

**Published:** 2017-11-24

**Authors:** Yanymee N. Guillen Quispe, Seung Hwan Hwang, Zhiqiang Wang, Guanglei Zuo, Soon Sung Lim

**Affiliations:** 1 Department of Food Science and Nutrition, Hallym University, 1 Hallymdeahak-gil, Chuncheon 24252, Korea; estreyany@gmail.com (Y.N.G.Q.); isohsh@gmail.com (S.H.H.); wangzq01234@gmail.com (Z.W.); guangleizuo@foxmail.com (G.Z.); 2 College of Public Health, Hebei University, Baoding 071002, China; 3 Institute of Natural Medicine, Hallym University, 1 Hallymdeahak-gil, Chuncheon 24252, Korea; 4 Institute of Korean Nutrition, Hallym University, 1 Hallymdeahak-gil, Chuncheon 24252, Korea

**Keywords:** Peruvian herbal extracts, α-glucosidase, aldose reductase, screening, ultrafiltration, *Hypericum laricifolium* Juss.

## Abstract

This study investigates in vitro targets related to diabetes in 30 herbal extracts from Peru, for the first time, using α-glucosidase, aldose reductase (AR) inhibitory assays and 2,2-diphenyl-1-picrylhydrazyl (DPPH) and 2,2′-azino-bis(3-ethylbenzothiazoline-6-sulfonic acid) (ABTS) scavenging assays. Among the 30 herbal extracts, *Hypericum laricifolium* Juss. (HL) was the herb which showed more than 50% inhibition in all assays, presenting 97.2 ± 2.0%, 56.9 ± 5.6%, 81.9 ± 2.5%, and 58.8 ± 4.6% inhibition for the α-glucosidase, AR, DPPH, and ABTS assays, respectively. Finally, six bioactive compounds, namely, protocatechuic acid, chlorogenic acid, caffeic acid, kaempferol 3-*O*-glucuronide, quercetin, and kaempferol were identified in HL by offline high-performance liquid chromatography (HPLC). Quercetin exhibited the strongest inhibition in all enzyme assays and the strongest antioxidant activity. The results suggest that HL shows great potential for the complementary treatment of diabetes and its complications.

## 1. Introduction

According to the World Health Organization (WHO), the prevalence of diabetes continues to increase at an alarming rate. Globally, an estimated 346 million people are living with diabetes, figures that are predicted to have a severe impact on human health by 2025 [1]. Diabetes mellitus (DM) is a group of chronic metabolic diseases characterized by chronic hyperglycemia. This condition is caused by the reduction of insulin secretion and/or insulin resistance and is considered as the primary factor for the pathogenesis of long-term diabetic complications [2]. Thus, diabetes is associated with the long-term damage, dysfunction and failure of various organs, leading to a series of complications caused by the disruption of carbohydrate, protein and fat metabolism, and these complications include nephropathy, neuropathy, retinopathy, atherosclerosis, skin complications, and cardiac dysfunction [1,3]. Therapy for DM relies on several approaches, many of which comprise drug targets for type 2 diabetes. Moreover, various efforts have been made to obtain other effective and safe enzyme inhibitors from plant extracts to control diabetes [4]. There are different targets related to diabetes and its complications such as α-glucosidase, aldose reductase (AR), and free radicals. α-Glucosidase (EC 3.2.1.20) is an important enzyme that catalyzes the final step of carbohydrate digestion. The inhibition of this enzyme can delay the digestion and absorption of dietary carbohydrates and consequently suppress postprandial hyperglycemia [4,5]. AR (EC 1.1.1.21) is the first enzyme in the polyol pathway. The high blood glucose levels characteristic of DM cause a significant flux of glucose through the polyol pathway in tissues such as kidney, nerve, and retina tissue [6]. Consequently, the accumulation of sorbitol generates osmotic stress and can activate AR, resulting in various diabetic complications [7]. Oxidative stress causes an imbalance between the free-radical-generating and free-radical-scavenging capacities. This imbalance is mainly responsible for the auto-oxidation of glucose in DM and its complications. The increased free radical production and reduced antioxidant defense may partially mediate the initiation and progression of diabetes-associated complications [8]. Thus, α-glucosidase and AR inhibitors and strong antioxidants may be useful tools to decrease postprandial blood glucose and insulin levels in patients with type 2 diabetes, prevent the polyol pathway, and ameliorate oxidative stress, respectively [4,9].

Research over the past two centuries has led to the development of a significant number of pharmaceuticals derived from plants from different regions of the world such as the South American rainforests [10]. In Peru, various types of plants are produced and consumed on a large scale. However, literature and information on the antidiabetic activity of these plants (especially on α-glucosidase and AR inhibition), which may lead to the development of new antidiabetic agents, is limited. Thus, this study investigates the efficacy of 30 herbal extracts from Peru for α-glucosidase and AR inhibitors and antioxidants. *Hypericum laricifolium* Juss. (HL) is a species of *Hypericum* (Clusiaceae) that is widely distributed in high altitude tropical regions, particularly in South America. In Peru, it is called “Chinchango, Abrecaminos, Hierba de la fortuna”, while in Ecuador it is called “Matikillkana, Romerillo, Hierba de San Juan” and has been used as folk medicine [11]. Previous reports have revealed the presence of various xanthones [12], phenolic acids, flavonoids, triterpenoids [13], and acylphloroglucinol derivatives in HL [14].

Traditional methods comprising isolation, fractionation, purification, and structure elucidation have been widely used to discover new bioactive compounds with antioxidants, α-glucosidase, and AR inhibitory activities. However, these traditional methods are time-consuming, labor intensive, and of low efficiency due to the loss of compound activity during isolation and purification [15]. Thus, it is necessary to establish effective and rapid methods, such as various offline high-performance liquid chromatography (HPLC) assays, to identify active compounds from mixtures. These include offline α-glucosidase ultrafiltration-HPLC, offline AR ultrafiltration-HPLC, offline 2,2-diphenyl-1-picrylhydrazyl (DPPH)-HPLC and offline 2,2′-azino-bis (3-ethylbenzothiazoline-6-sulfonic acid) (ABTS)-HPLC assays.

To the best of our knowledge, no screening method has been applied to herbal extracts related to diabetes and no affinity reports based on offline HPLC assay have been reported for HL to date. Thus, this study uses innovative screening methods for 30 herbal extracts from Peru related to diabetes and subsequently, an ultrafiltration method and offline DPPH-HPLC and ABTS-HPLC assays to screen active compounds for HL.

## 2. Results and Discussion

### 2.1. Evaluation of α-Glucosidase and Aldose Reductase (AR) Inhibition and Antioxidant Activity of Peruvian Plants

In this study, an assortment of plant parts including leaves, aerial parts, seeds, and roots were used in the extraction of 30 plants obtained from a popular market in Lima. α-Glucosidase and AR inhibition and the antioxidant activity of the 30 Peruvian herbal extracts were investigated (Table 1). Acarbose, quercetin, l-ascorbic acid, and 6-hydroxy-2,5,7,8-tetramethylchroman-2-carboxylic acid (trolox) were used as positive controls. Of the 30 herbal extracts assayed, only 23 crude extracts exhibited inhibition at 500 μg/mL in the α-glucosidase inhibition assay. Among these extracts, 9 presented relatively high α-glucosidase inhibition (>50%). *Desmodium molliculum* (Kunth) A.P. De Candolle (P80), *Geranium dielsianum* R. Knuth (A35), and HL (A10) showed significantly (*p* < 0.05) higher α-glucosidase inhibition (≥80%) than those of others. Even though the α-glucosidase inhibition of HL was lower than P80 and A35, they are not significantly different. In the AR inhibition assay, only 23 crude extracts exhibited inhibition, with *Otholobium mexicanum* (L.f.) J.W. Grimes (A3), HL, *Flaveria bidentis* (L.) Kuntze (A31), *Tessaria integrifolia* Ruiz & Pav. (A17), *Gentianella tristicha* (Gilg) J.S. Pringle (P7), and *Argyrochosma nivea (Poir.)* Windham (A2), presenting significantly (*p* < 0.05) high AR inhibition (>50%) at 10 μg/mL. The crude extracts were assessed by DPPH and ABTS assays for their antioxidant capacities. A total of 13 extracts, namely A10, A35, *Phyllanthus niruri* L. (P5), Clinopodium brevicalyx (Epling) Harley & A. Granda (P11)*, Schkuhria pinnata* (Lam.) Kuntze ex Thell. (P14)*, Cheilanthes pilosa* Goldm. (P17)*, Eucalyptus globolus* Labill. (P39)*, Equisetum giganteum* L. (P55)*, Chuquiraga spinosa* Less. (P77)*,* *Tiquilia Paronychioides* (Phil.) Rich. (P36)*,* P80, *Gentianella tristicha* (Gilg) J.S. Pringle (P7) and *Peumus boldus Molina* (P40) presented relatively high antioxidant capacities (>50%) in both the DPPH assay at 143 μg/mL and the ABTS assay at 33 μg/mL, whereas only 19 crude extracts exhibited high antioxidant capacities (>50%) in the DPPH assay. HL (DPPH = 81.9%, ABTS = 58.8%), P36 (DPPH = 95.7%, ABTS = 97.1%), A35 (DPPH = 96.8%, ABTS = 83.9%), P5 (DPPH = 97%, ABTS = 99.6%), P11 (DPPH = 95.8%, ABTS = 94.1%), P14 (DPPH = 67.1%, ABTS = 71.4%), P39 (DPPH = 77.5%, ABTS = 99.5%), P40 (DPPH = 86.2%, ABTS = 95.7%) and P80 (DPPH = 89.9%, ABTS = 73.6%) showed significantly (*p* < 0.05) higher antioxidant activities than others. Even though the antioxidant capacity of HL was lower than those of A35, P5, P36, P80, P11, and P40 these latter samples displayed low inhibitory activity in the AR assay (<35%). These results suggested that HL was the most active sample in all assays and that HL should be further investigated.

Previous studies on the effect of methanol (MeOH) extracts of *Geranium dielsianum* R. Knuth (A35) on DM in the Peruvian Andes have shown a suppression in blood glucose elevation that has been attributed to inhibitory activity on α-glucosidase [16]. Moreover, some *Geranium* species are sold as antidiabetics in markets in Lima, Peru [17]. Another study reported *Eucalyptus globolus* L. (P39) was found to exhibit antioxidant activity against DPPH, ABTS, and B-carotene free radicals (75.6%, 81.60%, and 60.40%, respectively, at 50 μg/mL [18]. In Argentina, the branches and stems from *Equisetum giganteum* L. (P55) exhibited a great antioxidant activity [19]. There are no reports on the AR activity of these samples.

### 2.2. Effect of Hypericum laricifolium Juss. (HL) on α-Glucosidase and Aldose Reductase (AR) Inhibition and Antioxidant Activity

In our previous study, we demonstrated that HL, which is cultivated in various places in Peru at an altitude ranging between 2000 and 4500 m above sea level, exhibits tyrosinase activity [26]. In Peru, one study reported good effects on antiproliferative activities on human hepatocellular carcinoma Hep3B cells [21]. Previous studies have also reported that HL comprises caffeic acid, esters of long-chain aliphatic alcohols, sterols, triterpenoids, benzoic and cinnamic acid derivatives, flavonols, and flavonol glycosides. Moreover, quercetin and caffeic acid displayed anti-inflammatory activity through the inhibition of cyclooxygenase-1 and cyclooxygenase-2 [13,14]. In other studies, ethanol extracts from the leaves of HL exhibited inhibition against *Candida albicans* [29].

No information on α-glucosidase and AR in HL have been reported to date, but some publications mentioned that species of same genera (*Hypericum*) have been used in folk medicine for DM, such as the popular specie called *Hypericum perforatum* (St. John’s wort) [30,31]. *Hypericum perforatum* L., the most well-known of these species, whose main components were determined as flavonoid derivates, quercetin, kaempferol, isoquercetin, and rutin [29,30,32].

Following the results discussed in Section 2.1 and Section 2.2, we decided to further investigate the effect of HL on α-glucosidase, AR, and antioxidant activity by comparing the differences in α-glucosidase, AR, and antioxidant activity in nonpolar (methylene chloride (MC)) and polar (70% MeOH) solvent extracts at concentrations of 500 μg/mL for the α-glucosidase assay, 10 μg/mL for the AR assay, and 143 μg/mL and 33 μg/mL for the DPPH and ABTS assays, respectively. As illustrated in Table 2, the MC extract displayed low inhibition for all assays and thus, the IC_50_ values could not be determined. The 70% MeOH extract demonstrated good inhibition of α-glucosidase activity, with an inhibition rate of 92.36% at 500 μg/mL (IC_50_ = 56.6 μg/mL). These values were significantly (*p* < 0.05) higher than those observed for acarbose (55.82%; IC_50_ = 367.4 μg/mL). In the AR inhibition assay, the MeOH extract inhibited AR by 64.51% at 10 μg/mL (IC_50_ = 3.3 μg/mL), a value significantly (*p* < 0.05) lower than that observed for quercetin (83.7%; IC_50_ = 1.3 μg/mL). For the antioxidant capacities, HL inhibited DPPH radical by 93% at a concentration of 143 μg/mL (IC_50_ = 42.5 μg/mL) and ABTS radicals by 78.9% at 33 μg/mL (IC_50_ = 14.4 μg/mL). L-Ascorbic acid and trolox displayed IC_50_ values of 17.6 and 4.6 μg/mL in the DPPH and ABTS assays, respectively; these values indicated higher activity than those observed for the HL MeOH extract. However, even with these results, HL can still be considered as having a significant inhibitory effect on DPPH and ABTS free radicals. Following these results, a 70% MeOH extract was selected to determine the constituents responsible for antidiabetic activity. A study by Levent et al. [30] investigating MeOH and ethyl acetate extracts from *Hypericum perforatum* L. revealed that at a concentration of 200 μg/mL, the MeOH extract exhibited higher DPPH antioxidant activity than the ethyl acetate extract. This demonstrated that the MeOH extract was more effective in reducing DPPH radicals.

### 2.3. Identification of the Major Bioactive Components in Hypericum laricifolium Juss. (HL) by Offline High-Performance Liquid Chromatography (HPLC)

The concept of offline HPLC-based activity profiling has been proposed and implemented for the effective screening of bioactive compounds in natural product extracts [33]. Recently, many offline HPLC-based assays have been introduced and developed to provide new and rapid analytical methods and many studies have reported the successful application of ultrafiltration-HPLC, DPPH-HPLC, and ABTS-HPLC to identify bioactive components from complex extracts.

#### 2.3.1. Identification of the Bioactive Compounds in HL by α-Glucosidase Ultrafiltration Combined with HPLC

**Figure 1:**
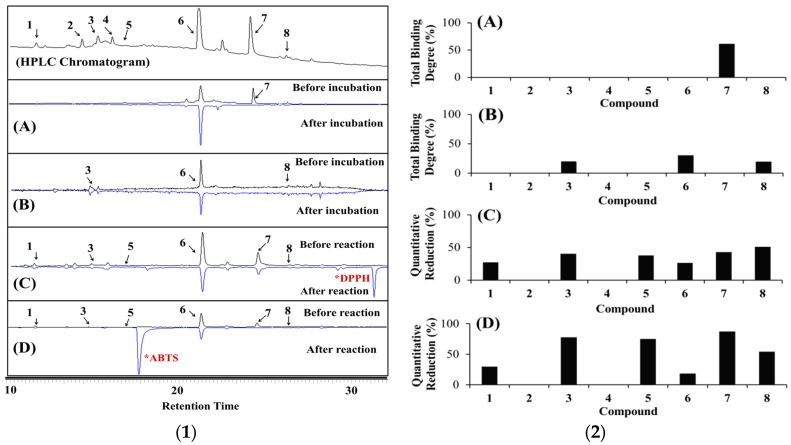
(**1**) HPLC chromatogram of crude *Hypericum laricifolium* Juss. (HL) 70% MeOH extract at 210 nm and offline HPLC of (**A**) HL based on offline α-glucosidase UF-HPLC. (**B**) HL based on offline HRAR UF-HPLC. Antioxidants determined by (**C**) offline DPPH-HPLC and (**D**) offline ABTS-HPLC. (**2**) Total Binding Degree (%) and Quantitative Reduction (%) of (**A**) offline α-glucosidase UF-HPLC, (**B**) offline HRAR UF-HPLC, (**C**) offline DPPH-HPLC and (**D**) offline ABTS-HPLC. * DPPH peak; * ABTS peak. HPLC: High-Performance Liquid Chromatography; UF: Ultrafiltration; HRAR: Human recombinant aldose reductase; DPPH: 2,2-diphenyl-1-picrylhydrazyl; ABTS: 2,2′-azino-bis(3-ethylbenzothiazoline-6-sulfonic acid); MeOH: Methanol.

**Figure 2:**
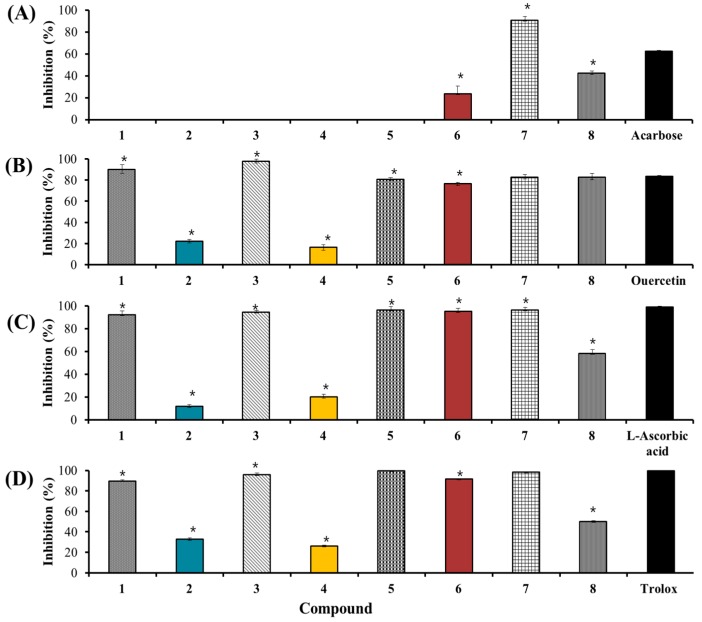
Inhibitory effects of the compounds isolated from *Hypericum laricifolium* Juss.: (**A**) α-glucosidase inhibitory activity; (**B**) aldose reductase (AR) inhibitory activity; (**C**) 2,2-diphenyl-1-picrylhydrazyl (DPPH) radical scavenging activity; (**D**) 2,2′-azino-bis(3-ethylbenzothiazoline-6-sulfonic acid) (ABTS) radical scavenging activity. Acarbose was used as positive control for the α-glucosidase assay, quercetin as positive control for the aldose reductase assay, l-ascorbic acid as positive control for the DPPH assay, and trolox as positive control for the ABTS assay. * The asterisk indicates a significant difference compared to positive control group (*p* < 0.05).

An ultrafiltration-HPLC assay was performed in this study to identify the potential α-glucosidase inhibitors in HL. The ultrafiltration-HPLC assay is an affinity-based screening method for bioactive compounds in a complex matrix whereby the ligands bind to the enzyme and are separated from unbound small molecules by the ultrafiltration membrane [34]. Thus, examination of the HPLC pattern of the decreased peaks indicates which ligands would be suitable as inhibitors. This pattern represents the ligands that bind to the enzyme, with a higher binding degree indicating compounds with higher binding affinities. Figure 1(1A,2A) illustrates that compound **7** exhibited a decrease in the peak area with a total binding degree (TBD) of 61.57% when the α-glucosidase ultrafiltration-HPLC system was applied, indicating that this compound is a strong α-glucosidase inhibitor. This is confirmed by its percentage inhibition (91%) and IC_50_ (15.9 μM) values (Figure 2 and Table 3, respectively). Compounds **1**–**6** and **8** were inactive.

#### 2.3.2. Identification of the Bioactive Compounds in HL by Aldose Reductase (AR) and Human Recombinant AR (HRAR) Ultrafiltration Combined with HPLC

For the HRAR ultrafiltration-HPLC assay (Figure 1(1B,2B)), three compounds exhibited decreases in peak areas: compound **3** displayed a decrease in peak area with a TBD of ~20%; compound **6** a decrease with a TBD of 30%, and compound **8** a decrease with a TBD < 20%. These values indicate that compounds **3** and **6** were the most potent AR inhibitors. Subsequently, an AR inhibitory assay was performed to confirm their activity. The IC_50_ values of compounds **1**, **3**, **5**, **6**, **7**, and **8** toward AR were 16.9 μM, 0.23 μM, 28.3 μM, 6.0 μM, 2.5 μM, and 9.7 μM, respectively, while compounds **2** and **4** were inactive (Figure 2 and Table 3). These values suggest that both compound **3** (0.23 μM) and compound **7** (2.5 μM) are strong AR inhibitors. The disappearance of some peaks after incubation was attributed to the low concentration of some components. However, in the case of quercetin, this was attributed to its solubility, binding affinities, and binding sites [34].

#### 2.3.3. Identification of Antioxidants Using Offline 2,2-Diphenyl-1-Picrylhydrazyl (DPPH)-(HPLC) and 2,2′-Azino-Bis (3-Ethylbenzothiazoline-6-Sulfonic Acid) (ABTS)-(HPLC)

A DPPH-HPLC/ABTS-HPLC approach was employed to identify the potential antioxidants in HL. In this simple and rapid approach, the extract was spiked with DPPH/ABTS. The peak areas of the antioxidants decreased or disappeared from the HPLC chromatogram after spiking with DPPH/ABTS, while almost no changes in peak areas were observed for components that did not exhibit any antioxidant activities. Thus, the more significant the reduction in peak area, the higher the antioxidant activity of the component. In the present study, the peaks from the DPPH-HPLC analysis of compounds **1**, **3**, **5**, **6**, **7**, and **8** decreased (Figure 1(1C,2C)) after the reaction, indicating that these compounds are antioxidants. The peak area reduction was quantitatively analyzed with % quantitative reduction (QR) values ranging between 20% and 50%. Moreover, compounds **7** and **8** displayed greater quantitative reductions (42.8% and 51% respectively), suggesting that these two compounds are more potent antioxidants than compounds **1**, **3**, **5**, and **6**. The IC_50_ values of compounds **1**, **3**, **5**, **6**, **7**, and **8** in the DPPH assay were determined as 263.4 μM, 123.1 μM, 47.2 μM, 58.8 μM, 0.3 μM, and 326.4 μM, respectively, whereas compounds **2** and **4** were inactive (Figure 1 and Table 3).

For the identification of antioxidants using ABTS-HPLC (Figure 1(1D,2D)), the ABTS-HPLC peaks of compounds **1**, **3**, **5**, **6**, **7**, and **8** decreased after the reaction, confirming the inhibition capacity of these compounds. Compound **7** presented a quantitative reduction percentage > 80%. This was followed by compounds **3** (77%), **5** (76%), and **8** (54%). The IC_50_ values of compounds **1**, **3**, **5**, **6**, **7**, and **8** in the ABTS assay were determined as 9.7 μM, 23.1 μM, 7.2 μM, 11.0 μM, 0.3 μM, and 191.8 μM, respectively; compound **7** exhibited the strongest activity while compounds **2** and **4** were inactive. From these observed results, we concluded that the % QR values in offline ABTS-HPLC were stronger than the corresponding values in offline DPPH-HPLC. This was attributed to the faster reaction kinetics and greater response to antioxidants of the ABTS assay that increased its sensitivity towards antioxidant activity [35]. Kaempferol and quercetin displayed higher quantitative reduction percentages in both offline DPPH-HPLC and ABTS-HPLC analyses.

Some studies have reported that members of the genus *Hypericum*, especially *H. perforatum* L., exhibit antioxidant effects as a result of their flavonoid and phenolic acid compositions [36]. Moreover, it has been reported that chlorogenic acid is the most active compound that contributes to the DPPH radical scavenging activity of *H. perforatum* L. [37]. In our study, chlorogenic acid also displayed antioxidant activity for DPPH and ABTS radicals, but was not the strongest antioxidant, probably due to its low concentration. On the other hand, it associated the antioxidant activity and α-glucosidase inhibitory properties of two species of *Hypericum,* namely *H. humifusum* and *H. perfoliatum*, with hypericin and hyperforin [38].

## 3. Materials and Methods

### 3.1. Materials

Nicotinamide adenine dinucleotide 2′-phosphate reduced tetrasodium salt hydrate (NADPH), 1Mm*p*-nitrophenyl-α-d-glucopyranoside, DL-glyceraldehyde dimer, DPPH, trolox, ABTS diammonium salt, aminoguanidine, acarbose, quercetin, and l-ascorbic acid were obtained from Sigma-Aldrich Chemical Co. (St. Louis, MO, USA). Human recombinant AR (HRAR) was obtained from Wako Pure Chemical Industries Co. (Osaka, Japan). Sephadex LH-20 was obtaimed from GE Healthcare Co. (Uppsala, Sweden). All organic solvents were obtained from J.T. Baker Co. (Phillipsburg, NJ, USA). All other reagents were obtained from Sigma-Aldrich Chemical Co. (St. Louis, MO, USA). Ultrapure water, used for dilutions and HPLC analysis, was obtained using a Milli-Q laboratory water purification system with a resistivity over 18.2 MΩ cm (Millipore Co., Bedford, MA, USA).

### 3.2. Collection of Plant Material

The plant materials used in this study, 30 dried Peruvian plants, were purchased from Mercado Aviacion in Lima, Peru (October 2015). The samples were identified using available literature in English and Spanish and verified by Paul H. Gonzales Arce from the Museo de Historia Natural, Universidad Nacional Mayor de San Marcos, Lima, Peru. The voucher specimens were deposited at the Center for Efficacy Assessment and Development of Functional Foods and Drugs, Hallym University, Korea. Plant names were verified on www.theplantlist.org in July 2015. Information on species name, family, plant organ and traditional uses is given in Table 1.

### 3.3. Preparation of Extracts and Isolation of Plant Samples

**Figure 3:**
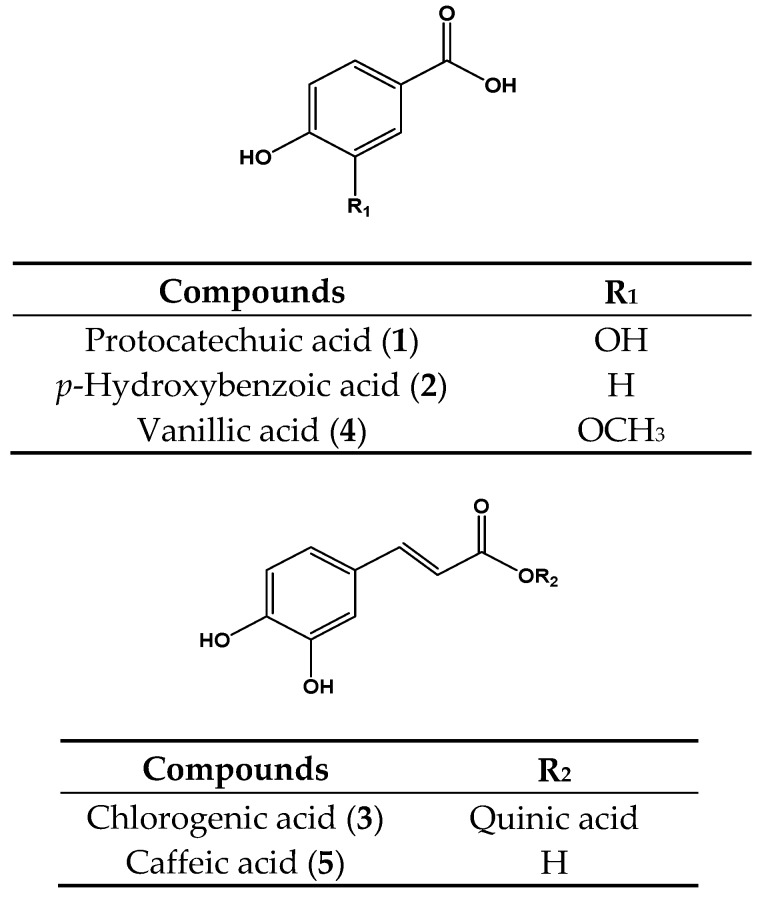

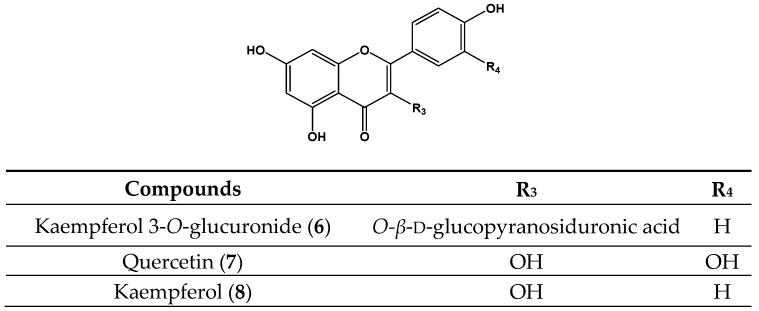
Chemical structures of the constituents isolated from *Hypericum laricifolium* Juss.

The 30 dried Peruvian plants (50 g) were pulverized and extracted by maceration at room temperature with 70% MeOH for 72 h. The yield for each extract, after solvent removal, was determined and expressed as a percentage of the dry weight of the plant parts used such as leaves, root, seed, fruit and aerial part. The major compounds of the HL 70% MeOH extract were isolated by column chromatography Sephadex LH-20 column [26]. In total, eight major compounds were identified: protocatechuic acid (**1**), *p*-hydroxybenzoic acid (**2**), chlorogenic acid (**3**), vanillic acid (**4**), caffeic acid (**5**), kaempferol 3-*O*-glucuronide (**6**), quercetin (**7**), and kaempferol (**8**) (Figure 3).

### 3.4. Evaluation of the α-Glucosidase Inhibitory Assay

α-Glucosidase inhibitory activity was determined using a previously reported method with slight modifications [39]. The initial concentration of the enzyme solution was 0.015 mg/mL in 0.1 M phosphate buffer (pH 6.9) and the initial concentration of the substrate solution was 1 mM in the same phosphate buffer. The enzyme solution was mixed in a clear 96-well microplate (flat bottom) and the reaction was initiated by the addition of the substrate to the solution. The plates were incubated at 37 °C for 10 min. Enzyme inhibition was determined from the absorbance of 4-nitrophenol (product) at 405 nm, measured with a microplate reader (EL800 Universal Microplate reader, BioTek instruments, Winooski, VT, USA). Acarbose was used as positive control. The percentage inhibition was calculated as follows: % inhibition α-glucosidase = [1 − (A_s_ − A_b_/A_c_ − A_b_)] × 100%, where A_c_ is the absorbance of the solution, buffer, enzyme, substrate (190 µL), and dimethyl sulfoxide (DMSO) (10 µL); A_b_ is the absorbance of the buffer solution (190 µL) with DMSO (10 µL); and A_s_ is the absorbance of the solution, buffer, enzyme, and substrate (190 µL) with the sample solution (10 µL). The results were also expressed as IC_50_ values (the concentration of sample required for 50% inhibition of enzyme activity).

### 3.5. Animal Care

All animal experiment procedures were conducted in accordance with the guidelines and approval of the Institutional Animal Care and Use Committees (IACUC) of Hallym University (Code: Hallym-2016-95; Date: 13 May 2016). The rat lenses (RL) were prepared as follows: the RL were removed from the eyes from 10-week-old Sprague-Dawley rats (weight = 250–280 g) and frozen until required. The rats were housed in a standardized laboratory environment with a 12 h light/12 h dark cycle at constant room temperature (22 ± 1 °C) and humidity (6 ± 5%) before experimentation. All animals had free access to water and food.

### 3.6. Evaluation of the Rat Lens Aldose Reductase (RLAR) Assay

The AR inhibitory activities were evaluated using RLAR. Prior to analysis, the lenses were homogenized in 0.1 M phosphate buffer saline (pH 6.2) and subsequently centrifuged at 10,000× *g* for 30 min at 4 °C. The rat lens aldose reductase (RLAR)-containing supernatant was then collected. A substrate comprising 531 μL 0.1 M potassium buffer (pH 7.0), 90 μL NADPH solution (1.6 mM in potassium buffer), 90 μL RLAR homogenate (6.5 U/mg), 90 μL ammonium sulfate solution (4 M in potassium buffer), and 90 μL DL-glyceraldehyde (25 mM in potassium buffer) was placed into a 1.0-mL cuvette and mixed with 9 μL sample (1 mg/mL dissolved in DMSO). RLAR activity was assessed spectrophotometrically by measuring the decrease in NADPH absorption at 340 nm over a 4 min period. Quercetin was used as positive control. The percentage inhibition was calculated as follows: % inhibition RLAR = [1 − (ΔA_s_ − ΔA_b_/ΔA_c_ − ΔA_b_)] × 100%, where ΔA_s_ is the reduction of absorbance of the reaction mixture ΔA_c_ is the same, but without the sample; and ΔA_b_ is the reduction of absorbance of the reaction mixture without the RLAR homogenate. The results were also expressed as IC_50_ values (the concentration of sample required for 50% inhibition of enzyme activity).

### 3.7. Evaluation of the DPPH Assay

The DPPH free radical scavenging activity was determined as previously described with slight modifications [34]. Briefly, 180 µL of DPPH solution (0.32 mM in methanol) was mixed with 30 µL of each sample at different concentrations (1.0–5.0 mg/mL in methanol). After 15 min incubation in the darkroom, the absorbance change at 570 nm was measured on a microplate reader (EL800 Universal Microplate reader, BioTek Instruments, Winooski, VT, USA). L-Ascorbic acid was used as positive control. The DPPH radical scavenging activity was calculated as % inhibition using the formula: % DPPH inhibition = [1 − (A_s_ − A_b_/A_c_ − A_b_)] × 100%, where A_c_ is the absorbance of the DPPH solution (180 µL) with methanol (30 µL); A_b_ is the absorbance of distilled water (180 µL) with methanol (30 µL); and A_s_ is the absorbance of DPPH solution (180 µL) with sample solution (30 µL). The results were also expressed as IC_50_ values (the concentration of sample required for inhibition of 50% DPPH radicals).

### 3.8. Evaluation of the ABTS Assay

The ABTS free radical scavenging activity was determined using ABTS radical cations (ABTS^+^) with slight modifications [34]. Briefly, potassium persulfate (3.5 mM) and ABTS diammonium salt (2.0 mM) were mixed, diluted in distilled water, and subsequently allowed to stand in the dark at room temperature for 24 h before use, to allow free radical generation. To determine the scavenging activity, the absorbance was recorded at 750 nm using an EL-800 Universal microplate reader (BioTek Instrument Inc., Winooski, VT, USA) after incubating the sample solutions (10 µL, 1 mg/mL, 0.5 mg/mL in methanol) with ABTS solution (290 µL) for 10 min in a 96-well plate. Trolox was used as a positive control. The ABTS radical scavenging activity was expressed as % inhibition using the following formula: % ABTS inhibition = [1 − (A_s_ − A_b_/A_c_ − A_b_)] × 100%, where A_c_ is the absorbance of the ABTS solution (290 µL) with methanol (10 µL); A_b_ is the absorbance of distilled water (290 µL) with methanol (10 µL); and A_s_ is the absorbance of the ABTS solution (290 µL) with the sample solution (10 µL). The results were also expressed as IC_50_ values (the concentration of sample required for the inhibition of 50% ABTS radicals).

### 3.9. Determination of the α-Glucosidase Ultrafiltration-HPLC Assay

The α-glucosidase present in the HL was profiled using a newly developed α-glucosidase ultrafiltration assay. Specifically, the HL 30 μL sample (final concentration = 0.1 mg/mL) was incubated with α-glucosidase (3.9 μM) in PBS (300 μL, pH 7.0) at 37 °C for 30 min. The incubated mixture was then filtered by centrifugation at 5167× *g* for 30 min at room temperature using a Microcon YM-10 centrifugal filter unit (Millipore, Bedford, MA, USA). The filtrate was subsequently analyzed by HPLC on a Dionex system (Dionex, Sunnyvale, CA, USA) consisting of a P850 pump, an ASI-100 automated sample injector, a Synergi Hydro-RP 80A column (150 mm × 4.6 mm, 4 μm; Phenomenex, Torrance, CA, USA) maintained at 30 °C, and a UVD170S detector. The injection volume was 10 μL and the flow rate of the mobile phase was 0.7 mL/min. The mobile phase compositions were as follows: 0.1% trifluoroacetic acid (A-line) and methanol (B-line). The gradient elution system was modified as follows: 0–35 min, starting with 5% B-line, programmed to reach 40% B-line at 15 min using a linear gradient, followed by 100% B-line from 15–35 min. The detector monitored the eluent at a wavelength of 254 nm. The sample incubated without α-glucosidase was used as control. The relative binding affinity of the inhibitors in the extract sample toward α-glucosidase was defined as the “total binding degree (TBD)”, calculated from the equation: TBD (%) = (A − B)/A × 100%, where A and B are the HPLC peak areas of a compound interacting without and with α-glucosidase, respectively.

### 3.10. Determination of the HRAR Ultrafiltration-HPLC Assay

The AR Inhibitors (ARIs) present in HL were profiled using a newly developed AR ultrafiltration assay. Specifically, the HL sample (final concentration = 0.1 mg/mL) was incubated with 0.6 M ammonium sulfate and 3.9 μM HRAR (Human recombinant AR) in a total volume of 300 μL at 37 °C for 30 min. The incubated mixture was then filtered by centrifugation at 5167× *g* for 30 min at room temperature using a Microcon YM-10 centrifugal filter unit. The filtrate was subsequently analyzed by HPLC (Section 3.9). The sample incubated without HRAR was used as control.

### 3.11. Evaluation of Offline DPPH-HPLC Analysis

The DPPH radical assay was modified by an offline DPPH-HPLC assay [40]. Briefly, 180 μL DPPH solution (0.32 mM in MeOH) was prepared and mixed with 30 μL 70% MeOH extract of HL (5 mg/mL in MeOH). After incubation (15 min) in the dark room, the mixture was filtered through a 0.45-μm membrane and subsequently analyzed by HPLC (Section 3.9). The sample incubated with methanol was used as control. The decreases in peak area were expressed as the % quantitative reduction (% QR), calculated from the equation: % QR = (A − B)/A × 100%, where A and B are the HPLC peak areas of the compound incubated without and with the DPPH methanol solution, respectively.

### 3.12. Evaluation of Offline ABTS-HPLC Analysis

Offline ABTS-HPLC was investigated by modifying a previously reported protocol [41]. Briefly, 10 μL of 70% methanol extract from HL (20 mg/mL) was mixed with 140 μL ABTS solution that was prepared one day in advance. The mixture was then incubated at room temperature for 10 min and subsequently filtered through a 0.45 μm filter. The filtrate was analyzed by HPLC (Section 3.9). The methanol extract of HL (20 mg/mL in MeOH was used as control. The detector monitored the eluent at a wavelength of 300 nm and the % QR was calculated as described in Section 3.11.

### 3.13. Statistical Analysis

The mean values were calculated based on at least three independent replicates (*n* = 3) and the results are expressed as mean ± standard deviations (SD). All data were statistically analyzed by one-way analysis of variance (ANOVA) followed by Tukey’s test and Dunnet’s test using the SPSS software package, IBM SPSS Statistics 20 (SPSS Inc., Chicago, IL, USA). A value of *p* < 0.05 was considered statistically significant.

## 4. Conclusions

In this study, α-glucosidase, AR, and antioxidant test screening of 30 Peruvian herbal extracts illustrated that HL exhibited the greatest activity in all assays. Consequently, compounds such as quercetin, present in HL were identified by α-glucosidase ultrafiltration-HPLC, while chlorogenic acid, kaempferol 3-*O*-glucuronide, and kaempferol were identified by AR ultrafiltration-HPLC. Protocatechuic acid, chlorogenic acid, caffeic acid, kaempferol 3-*O*-glucuronide, quercetin, and kaempferol were identified as antioxidants by off-line DPPH and ABTS-HPLC. The results suggest that HL shows great potential for the complementary treatment of diabetes and its complications.

## Figures and Tables

**Table 1:** Ethnobotanical data related to diabetes, treatment, complications, symptoms, obesity, infection, inflammation and α-glucosidase, aldose reductase (AR) inhibitory activities, and antioxidants of Peruvian herbal extracts.

No.	Voucher	Scientific Name	Common Name	Family	Used Part ^1^	References [13,17,19,20,21,22,23,24,25,26,27,28] ^2^	Yield (%) ^3^	Inhibition (%) ^4^			
								α-Glucosidase (500 μg/mL) ^5^	AR (10 μg/mL) ^6^	Antioxidants	
										DPPH (143 μg/mL) ^7^	ABTS (33 μg/mL) ^8^
1	A5	*Annona muricata* L.	Hoja de huanabana, Graviola	ANNONACEAE	L	Hypertension [ 24], Inflammation [20,24]	19.5	26.3 ± 1.7 ^c^	0.8 ± 0.8 ^a^	64.2 ± 0.9 ^e,f,g,h,i^	31.5 ± 2.9 ^f,g^
2	A32	*Acanthoxanthium spinosu* (L.) Fourr.	Juan Alonso	ASTERACEAE	A	Diabetes [ 25], Inflammation [20]	8.4	3.2 ± 2.7 ^a^	22.4 ± 1.2 ^e,f^	11.0 ± 3.5 ^a,b^	2.1 ± 1.8 ^a^
3	A16	*Ambrosia arborescens* Mill.	Marco	ASTERACEAE	L	Inflammation [ 25]	12.7	18.5 ± 1.9 ^c^	31.8 ± 6.1 ^f,g^	3.5 ± 0.7 ^a,b^	6.1 ± 1.8 ^a,b^
4	P78	*Baccharis genistelloides* (Lam.) Pers.	Karqueja	ASTERACEAE	A	Diabetes [ 20,21], Inflammation [20], Burn fat [20], Cholesterol [20,21]	19.7	6.2 ± 3.4 ^a,b^	NA	47.0 ± 9.2 ^c,d^	27.9 ± 1.1 ^e,f,g^
5	P77	*Chuquiraga spinosa* Less.	Huamanpinta	ASTERACEAE	A	Inflammation [ 25], Kidneys [25]	28.7	NA ^9^	12.2 ± 0.7 ^c,d^	70.3 ± 11.0 ^g,h,i,j^	50.3 ± 3.1 ^h^
6	A31	*Flaveria Bidentis* (L.) Kuntze	Mata gusano	ASTERACEAE	L	Inflammation [ 26]	7.9	17.3 ± 8.4 ^b,c^	86.4 ± 1.9 ^k^	5.3 ± 10.8 ^a,b^	8.0 ± 2.1 ^a,b^
7	P14	*Schkuhria pinnata* (Lam.) Kuntze ex Thell.	Canchalagua	ASTERACEAE	A	Diabetes [ 20,21], Inflammation [20,21]	2.4	0.1 ± 2.7 ^a^	NA	67.1 ± 3.3 ^f,g,h,i^	71.4 ± 8.4 ^j^
8	A6	*Smallanthus sonchifolius* (Poepp.) H. Rob.	Hojas de yacon	ASTERACEAE	L	Diabetes [ 20,21,22,23], Inflammation [20], Kidneys [20], Cholesterol [20]	5.3	5.5 ± 1.4 ^a,b^	31.9 ± 9.1 ^g^	NA	5.5 ± 3.0 ^a,b^
9	P49	*Taraxacum officinale* F.H. Wigg.	Diente de leon	ASTERACEAE	A, F	Inflammation [ 20]	4.8	NA	NA	41.9 ± 10.8 ^c^	15.7 ± 1.6 ^b,c,d^
10	A17	*Tessaria integrifolia* Ruiz & Pav.	Pajaro bobo	ASTERACEAE	L	Kidneys [ 20], Liver [20], Inflammation [20]	18.4	41.6 ± 1.1 ^d^	79.9 ± 0.1 ^k^	52.2 ± 3.9 ^c,d,e^	31.1 ± 3.3 ^f,g^
11	P79	*Cordia lutea* Lam.	Flor de overo	BORAGINACEAE	F	Inflammation [ 20,21], Liver [20,21]	12.3	NA	3.6 ± 1.3 ^a,b^	60.2 ± 2.1 ^d,e,f,g,h^	36.6 ± 1.7 ^g^
12	P36	*Tiquilia Paronychioides* (Phil.) Rich.	Flor de arena	BORAGINACEAE	A	Inflammation [ 20]	18.5	2.9 ± 1.1 ^a^	26.0 ± 0.1 ^f,g^	95.7 ± 0.8 ^m,n^	97.1 ± 0.4 ^m^
13	A24	*Sambucus peruviana* H. B. K.	Sauco	CAPRIFOLIAEAE	L	Kidneys [ 20,23], Inflammation [20]	8.1	47.4 ± 4.8 ^d^	47.2 ± 0.9 ^h^	53.3 ± 1.5 ^c,d,e,f^	33.8 ± 6.1 ^g^
14	A10	*Hypericum laricifolium* Juss.	Hierba de la fortuna	CLUSIACEAAE	L	Inflammation [ 13,28], Infections, Musculoskeletal, Bone pain [19]	15.9	97.2 ± 2.0 ^h^	56.9 ± 5.6 ^i^	81.9 ± 2.5 ^j,k,l,m^	58.8 ± 4.6 ^h,i^
15	P55	*Equisetum giganteum* L.	Cola de caballo	EQUISETACEAE	A	Kidneys [ 20,21], Inflammation [20,21]	11.2	77.8 ± 5.9 ^g^	0.7 ± 0.0 ^a^	73.9 ± 8.9 ^h,i,j,k^	51.8 ± 0.3 ^h^
16	P5	*Phyllanthus niruri* L.	Chanca piedra	EUPHORBIACEAE	L	Diabetes [ 24], Kidneys [20], Liver [20], Inflammation [20]	12.9	39.9 ± 1.4 ^d^	25.3 ± 3.7 ^f,g^	97.0 ± 0.1 ^n^	99.6 ± 0.1 ^m^
17	P80	*Desmodium molliculum* (Kunth) A.P. De Candolle	Manayupa	FABACEAE	L	Kidneys [ 20,21], Inflammation [20,21]	23.8	97.9 ± 9.1 ^h^	10.9 ± 0.5 ^b,c,d^	89.9 ± 4.2 ^l,m,n^	73.6 ± 1.3 ^j,k^
18	A3	*Otholobium mexicanum* (L. f.) J.W. Grimes	Culen negro	FABACEAE	A	Diabetes [ 21]	6.1	NA	50.4 ± 0.5 ^h,i^	4.5 ± 5.7 ^a,b^	9.6 ± 1.9 ^a,b,c^
19	A4	*Otholobium pubescens* (Poir.) J.W. Grimes	Culen blanco	FABACEAE	A	Diabetes [ 21]	19.6	NA	8.8 ± 0.5 ^a,b,c,d^	6.1 ± 0.5 ^a,b,c,d^	27.4 ± 1.5 ^e,f,g^
20	A49	*Vicia Faba*	Haba	FABACEAE	Fr	Renal disorders [ 22]	7.6	0.1 ± 2.7 ^a^	NA	4.2 ± 2.0 ^a,b^	13.0 ± 3.0 ^b,c,d^
21	P7	*Gentianella tristicha (Gilg)* J.S. Pringle	Hercampure	GENTIANACEAE	A	Diabetes [ 17], Cholesterol [26]	30.3	NA	68.1 ± 4.2 ^j^	53.1 ± 0.6 ^cdef^	66.5 ± 4.5 ^i,j^
22	A35	*Geranium dielsianum* R. Knuth	Pasuchaca	GERANIACEAE	L	Diabetes [ 17,24]	6.3	97.7 ± 1.1 ^h^	15.6 ± 0.2 ^d,e^	96.8 ± 0.3 ^n^	83.9 ± 4.1 ^k,l^
23	P11	*Clinopodium brevicalyx* (Epling) Harley & A. Granda	Inka muña	LAMIACEAE	L	Inflammation [ 26]	26.8	1.8 ± 5.5 ^a^	31.9 ± 0.7 ^g^	95.8 ± 0.2 ^m,n^	94.1 ± 2.7 ^l,m^
24	P4	*Salvia hispanica* L.	Chia	LAMIACEAE	S	Diabetes [ 27], Obesity [27]	3.5	NA	NA	17.3 ± 3.0 ^b^	21.7 ± 1.8 ^d,e,f^
25	P40	*Peumus boldus* Molina	Boldo	MONIMIACEAE	L	Inflammation [ 20], Kidneys [20], Liver [20]	32.5	75.2 ± 3.0 ^g^	7.6 ± 0.9 ^a,b,c,d^	86.2 ± 3.9 ^k,l^^,^^m,n^	95.7 ± 0.5 ^m^
26	P39	*Eucalyptus globolus* Labill.	Eucalipto	MYRTACEAE	L	Burn fat [ 20]	18.0	62.3 ± 0.9 ^e,f^	11.2 ± 0.8 ^b,c,d^	77.5 ± 0 ^i,j,k,l^	99.5 ± 0.3 ^m^
27	A2	*Argyrochosma nivea* (Poir.) Windham	Cuti - Cuti hembra blanca	PTERIDACEAE	A	Diabetes [ 17]	2.3	50.5 ± 3.2 ^d,e^	59.1 ± 0.3 ^i,j^	71.2 ± 0.7 ^g,h,i,j^	34.7 ± 7.3 ^g^
28	P17	*Cheilanthes pilosa* Goldm.	Cuti Cuti	PTERIDACEAE	A	Diabetes [ 26], Liver [26]	14.3	72.4 ± 0.6 ^f,g^	9.6 ± 0.9 ^b,c,d^	58.9 ± 4.8 ^d,e,f,g^	56.4 ± 4.3 ^h,i^
29	A1	*Cheilanthes pruinata* Kaulf.	Cuti - Cuti marron macho	PTERIDACEAE	A	Diabetes [ 17]	30.3	73.4 ± 2.9 ^f,g^	41.6 ± 1.1 ^h^	53.2 ± 0.3 ^c,d,e,f^	31.1 ± 4.1 ^f,g^
30	A19	*Buddleja Americana* L.	Flor blanca	SCROPHULARIA CEAE	F	Inflammation [ 26]	8.5	16.1 ± 5.6 ^b,c^	3.7 ± 0.1 ^a,b,c^	2.4 ± 1.0 ^a^	19.6 ± 1.2 ^c,d,e^

^1^ L, leaf; A, arial part; F, flowers; Fr, fruits; S, seed. ^2^ Information on medicinal uses and previous publications, [13,17,19,20,21,22,23,24,25,26,27,28]. ^3^ Yield (%) was calculated as weight of air-dried crushed plant material with respect to the starting material. ^4^ Inhibition (%) was provided as the mean ± standard deviation. ^a^^–^^n^ Different letters in the same column indicate significant differences, *p* < 0.05. ^5^ α-Glucosidase inhibitory activities. Positive control, acarbose (54.2 ± 3.6% Inhibition). ^6^ AR inhibitory activities. Positive control, quercetin (88.1 ± 1.6% Inhibition). ^7^ Antioxidant activity of DPPH radical scavenging. Positive control, l-ascorbic acid (98.9 ± 0.6% Inhibition). ^8^ Antioxidant activity of ABTS radical scavenging. Positive control, trolox (97.2 ± 2.0% Inhibition). ^9^ NA is not active. AR: Aldose reductase; DPPH: 2,2-diphenyl-1-picrylhydrazyl; ABTS: 2,2′-azino-bis(3-ethylbenzothiazoline-6-sulfonic acid).

**Table 2:** Inhibitory activities and IC_50_ values (µg/mL) of *Hypericum laricifolium* Juss. (HL) non-polar and polar extracts against α-glucosidase, aldose reductase (AR), and antioxidants.

Extracts	α-Glucosidase ^1^		AR ^2^		DPPH ^3^		ABTS ^4^	
	Inhibition (%)	IC_50_ (μg/mL) ^5^	Inhibition (%)	IC_50_ (μg/mL)	Inhibition (%)	IC_50_ (μg/mL)	Inhibition (%)	IC_50_ (μg/mL)
**MC**	11.65 ± 2.1 *	-	28.9 ± 7.4 *	-	17.5 ± 0.3 *	-	14.4 ± 1.3 *	-
**70% MeOH**	92.36 ± 1.1 *	56.6 ± 2.5	64.51 ± 1.3 *	3.3 ± 52	93.0 ± 0.1 *	42.5 ± 0.6	78.9 ± 0.6 *	14.4 ± 1.3
**Acarbose**	55.82 ± 2.3	367.4 ± 2.1	-	-	-	-	-	-
**Quercetin**	-	-	83.7 ± 2.6	1.3 ± 3.6	-	-	-	-
**L-Ascorbic**	-	-	-	-	99.1 ± 0	17.6 ± 0.1	-	-
**Trolox**	-	-	-	-	-	-	100 ± 0.1	4.6 ± 0.1

^1^ The inhibition rates (%) were calculated at a concentration of 500 μg/mL. ^2^ The inhibition rates (%) were calculated at a concentration of 10 μg/mL. ^3^ The inhibition rates (%) were calculated at a concentration of 143 μg/mL except for that of l-ascorbic acid that was calculated at a concentration of 71 μg/mL. ^4^ The inhibition rates (%) were calculated at a concentration of 33 μg/mL except for that of trolox that was calculated at a concentration of 17 μg/mL. ^5^ IC_50_ is defined as the half-maximal inhibitory concentration and data is presented as means ± SD (*n* = 3). * The asterisk indicates a significant difference compared to positive control group (*p* < 0.05). MC: Methylene chloride; MeOH: Methanol.

**Table 3:** Inhibition and IC_50_ values (µM) for tyrosinase, α-glucosidase, and aldose reductase (AR) and antioxidant activities of isolated compounds from *Hypericum laricifolium* Juss.

Compounds	α-glucosidase ^1^		AR ^2^		Antioxidants			
					DPPH ^3^		ABTS ^4^	
	Conc. (μg/mL)	IC_50_ (μM) ^5^	Conc. (μg/mL)	IC_50_ (μM)	Conc. (μg/mL)	IC_50_ (μM)	Conc. (μg/mL)	IC_50_ (μM)
Protocatechuic acid (**1**)	50	NA ^6^	10	16.9 ± 1.9 ^e^	143	263.4 ± 0.2 ^e^	17	9.7 ± 0.9 ^c^
*p*-Hydroxybenzoic acid (**2**)	50	NA	10	NA	143	NA	33	NA
Chlorogenic acid (**3**)	50	NA	10	0.23 ± 1.8 ^a^	143	123.1 ± 2.9 ^d^	33	23.1 ± 3.5 ^f^
Vanilic acid (**4**)	50	NA	10	NA	143	NA	33	NA
Caffeic acid (**5**)	50	NA	10	28.3 ± 0.9 ^f^	143	47.2 ± 0.1 ^b^	17	7.2 ± 1.1 ^b^
Kaempferol 3-*O*-glucuronide (**6**)	50	NA	10	6.0 ± 1.1 ^c^	143	58.8 ± 2.1 ^c^	17	11.0 ± 0.2 ^d^
Quercetin (**7**)	50	15.9 ± 1.2 ^a^	10	2.5 ± 0.8 ^b^	71	0.33 ± 1.4 ^a^	17	0.33 ± 0.6 ^a^
Kaempferol (**8**)	50	NA	10	9.7 ± 0.5 ^d^	143	326.4 ± 3.3 ^f^	33	191.8 ± 1.1 ^g^
Acarbose	500	439.9 ± 8.9 ^b^	-	-	-	-	-	-
Quercetin	-	-	10	2.4 ± 1.2 ^b^	-	-	-	-
l-Ascorbic	-	-	-	-	71	0.57 ± 0.4 ^a^	-	-
Trolox	-	-	-	-	-	-	17	18.8 ± 0.5 ^e^

^1^ The inhibition rates (%) were calculated at a concentration of 50 μg/mL except that for that of acarbose that was calculated at a concentration of 500 μg/mL. ^2^ The inhibition rates (%) were calculated at a concentration of 10 μg/mL. ^3^ The inhibition rates (%) were calculated at a concentration of 143 μg/mL except for that of l-ascorbic acid that was calculated at a concentration of 71 μg/mL. ^4^ The inhibition rates (%) were calculated under the concentration of 17 μg/mL. ^5^ IC_50_ value is defined as the half-maximal inhibitory concentration and data is presented as means ± SD (*n* = 3). ^a–g^ Different letters in the same column indicate significant differences, *p* < 0.05. ^6^ NA is not active.

## Abbreviations

AR	Aldose reductase
DPPH	2,2-Diphenyl-1-picrylhydrazyl
ABTS	2,2′-Azino-bis(3-ethylbenzothiazoline-6-sulfonic acid)
DMSO	Dimethyl sulfoxide
HL	*Hypericum laricifolium* Juss.
MeOH	Methanol
RLAR	Rat lens aldose reductase
HRAR	Human recombinant aldose reductase
HPLC	High-performance liquid chromatography
TBD	Total binding degree
QR	Quantitative reduction
